# Self‐Powered Bio‐Inspired Spider‐Net‐Coding Interface Using Single‐Electrode Triboelectric Nanogenerator

**DOI:** 10.1002/advs.201900617

**Published:** 2019-05-29

**Authors:** Qiongfeng Shi, Chengkuo Lee

**Affiliations:** ^1^ Department of Electrical and Computer Engineering National University of Singapore 4 Engineering Drive 3 Singapore 117576 Singapore; ^2^ Center for Intelligent Sensors and MEMS National University of Singapore Block E6 #05‐11, 5 Engineering Drive 1 Singapore 117608 Singapore; ^3^ Hybrid‐Integrated Flexible (Stretchable) Electronic Systems Program National University of Singapore Block E6 #05‐3, 5 Engineering Drive 1 Singapore 117608 Singapore; ^4^ NUS Suzhou Research Institute (NUSRI) Suzhou Industrial Park Suzhou 215123 P. R. China; ^5^ NUS Graduate School for Integrative Science and Engineering National University of Singapore Singapore 117456 Singapore

**Keywords:** bio‐inspired interfaces, scalability, self‐powered electronics, single electrodes, triboelectric nanogenerators

## Abstract

Human–machine interfaces are essential components between various human and machine interactions such as entertainment, robotics control, smart home, virtual/augmented reality, etc. Recently, various triboelectric‐based interfaces have been developed toward flexible wearable and battery‐less applications. However, most of them exhibit complicated structures and a large number of electrodes for multidirectional control. Herein, a bio‐inspired spider‐net‐coding (BISNC) interface with great flexibility, scalability, and single‐electrode output is proposed, through connecting information‐coding electrodes into a single triboelectric electrode. Two types of coding designs are investigated, i.e., information coding by large/small electrode width (L/S coding) and information coding with/without electrode at a predefined position (0/1 coding). The BISNC interface shows high scalability with a single electrode for detection and/or control of multiple directions, by detecting different output signal patterns. In addition, it also has excellent reliability and robustness in actual usage scenarios, since recognition of signal patterns is in regardless of absolute amplitude and thereby not affected by sliding speed/force, humidity, etc. Based on the spider‐net‐coding concept, single‐electrode interfaces for multidirectional 3D control, security code systems, and flexible wearable electronics are successfully developed, indicating the great potentials of this technology in diversified applications such as human–machine interaction, virtual/augmented reality, security, robotics, Internet of Things, etc.

## 1 Introduction

Human–machine interfaces (HMIs) are essential components providing communication between the interactions of human and machine in diverse applications, e.g., entertainment, robotics, security, virtual reality/augmented reality (VR/AR), smart home, smart factory, etc. Evolving together with the mainstream electronic technologies, modern HMIs are developing toward two directions, i.e., flexible wearable compatibility and self‐powered capability. Recently, flexible wearable electronics are attracting more and more research interests from worldwide due to their unique and irreplaceable features of soft, light, comfortable, and wearable property.^1^, ^2^, ^3^, ^4^, ^5^, ^6^, ^7^ Accordingly, the developed flexible wearable electronics have been further applied for a large variety of applications, such as energy scavenging,^8^, ^9^, ^10^ motion detection,^11^, ^12^ physiological monitoring,^13^, ^14^, ^15^ physical/chemical sensing,^16^, ^17^ soft robotics,^18^, ^19^ healthcare,^20^, ^21^, ^22^ HMI,^23^, ^24^ etc. In this regard, flexible wearable HMIs can be comfortably worn on human body to enable smart control whenever and wherever possible. On the other hand, self‐powered electronics are also of increasing importance due to their self‐sustainability and energy saving nature, showing great potential to ease the global energy crisis and environment pollution. Many self‐powered electronics have been developed through the integration with different energy harvesting technologies, such as electromagnetic,^25^, ^26^ piezoelectric,^27^, ^28^, ^29^ triboelectric,^30^, ^31^, ^32^ etc. Since its first invention, triboelectric nanogenerator (TENG) has been proven as a promising energy harvesting technology, which receives rapid development of diverse energy harvesters and self‐powered sensors globally.^32^, ^33^, ^34^, ^35^, ^36^, ^37^, ^38^, ^39^, ^40^, ^41^ TENG technology provides an ideal approach to realize HMIs with both flexible wearable compatibility and self‐powered capability,^42^, ^43^, ^44^, ^45^, ^46^, ^47^ because of its superior merits including self‐generated signal, high output performance, diverse and simple device configuration, ultra‐wide material applicability, flexibility/stretchability, good scalability, and low cost.^48^, ^49^, ^50^, ^51^, ^52^, ^53^, ^54^, ^55^, ^56^

During the past few years, various triboelectric interfaces have been developed toward a wide range of detection and control applications.^57^, ^58^, ^59^ Categorized by the actuated motion performing on the interfaces, triboelectric interfaces mainly include pressing‐type interfaces and sliding‐type interfaces. Pressing‐type interface is normally realized by contact‐separation mode–based pressure or tactile sensors, in order to detect the pressing on the interface for motion control.^60^, ^61^, ^62^, ^63^, ^64^, ^65^, ^66^, ^67^, ^68^, ^69^ For each of the motion control, individual sensing node is required as well as sensing electrodes. To give an example, a flexible and transparent TENG interface using array of multiple tactile sensors is demonstrated for the detection and mapping of finger contact, which then can be further applied for gaming control.^64^ Another triboelectric interface in form of 3D tactile sensor is reported for intuitive control of object attitude in 3D manner (including both translation and rotation movement).^60^ The 3D tactile sensor in total has eight sensing nodes/electrodes to detect different operation motions (two finger pressing on both sides of the device) for 3D control. Although these developed pressing‐type interfaces exhibit relatively stable and high output performance, they require the designs of complicated structures and a large number of sensors/electrodes for different motion control.

On the other hand, sliding‐type interface is normally achieved by detecting the sliding motion with pressure/tactile sensor array or strip/grating electrodes along the trace.^58^, ^70^, ^71^, ^72^, ^73^, ^74^, ^75^ A 9 × 9 sensor array with separated electrodes is developed to achieve real‐time detection of contact position, trajectory, velocity, and acceleration of a sliding object.^70^ However, sensor array‐based interfaces have low scalability with the large number of electrodes. When implementing higher resolution, the number of sensors and electrodes needs to be increased dramatically, introducing significant difficulty in readout circuitry and signal processing. To reduce the number of electrodes, a triboelectric interface that is able to detect finger sliding trajectory with only four electrodes is proposed.^71^ Grid patterns are attached on the surface to convert the continuous finger sliding into periodic contract and separation along the trace. The four electrodes are located at the edges and the detection is based on the charge‐induced voltage ratio of opposite electrodes. Another sliding‐type interface is developed using strip electrodes with varying length, for the monitoring of vehicle moving direction, speed, and acceleration.^76^ Compared to pressing‐type interfaces and sensor array–based sliding‐type interfaces, strip electrode–based sliding‐type interfaces exhibit great advantages for multiple motion control, e.g., simple device configuration, high intuitiveness, and reduced number of electrodes. With less number of electrodes in interfaces, beneficial gains in device fabrication, signal detection, and processing without signal crosstalk can be obtained. The ultimate goal of reducing the number of electrodes is the minimalist design, i.e., single‐electrode interface.

Sliding‐type interfaces provide promising potential to realize single‐electrode interface through advanced structure innovation. A possible design is using connected strip electrodes with varying number of electrodes in different directions for multiple motion control. When sliding across the electrode patterns, output signal peaks with number as same as the number of the strip electrodes are generated. Thus in this way, different sliding directions can be distinguished and used to define different control motions. However, this design through distinguishing the number of output peaks exhibits low scalability. That is, if a large amount of control motions are required such as multidirectional control with good resolution, the last electrode pattern needs to have a large number of strip electrodes, which greatly increases the device dimension and causes significant imbalance in different directions (e.g., larger sliding length for patterns with larger number of strip electrodes). To overcome this issue, information coding can be potentially introduced into the sensing electrodes,^53^, ^76^, ^77^ then forming 2D layout for multidirectional detection and control.

Nowadays, bio‐inspired electronics especially the self‐powered bio‐inspired electronics have gradually evolved into one of the most important and emerging research areas,^78^, ^79^, ^80^, ^81^ developing toward diverse applications such as energy conversion,^82^ implanted electronics,^83^ electronic skin,^84^, ^85^ energy storage,^86^ sensors,^87^ etc. Here in this work, a spider‐net layout‐inspired single‐electrode triboelectric interface is developed through information coding on the sensing electrode. This bio‐inspired spider‐net‐coding (BISNC) interface is highly scalable, robust, and reliable for the signal detection in various scenarios, such as different sliding force, sliding speed, and ambient humidity, since the signal detection mechanism is independent of the absolute amplitude. Two types of information coding configurations are investigated to achieve unique and distinguishable output signal for each direction with the single‐electrode output. The first configuration is information coding with large/small electrode width, where the output patterns can be differentiated through the relative amplitude of output peaks. The second configuration is based on information coding with and/or without a strip electrode on a predefined position. Accordingly, the output signal patterns can be interpreted from the positions of the generated signal peaks in time domain. The advanced information coding concept enables the realization of highly scalable and single‐electrode triboelectric interfaces, toward diversified applications including 3D control, security, VR/AR, human–machine interaction, robotics, etc.

## 2 Operation Principle and Self‐Powered Characteristics of BISNC Interface

To realize multidirectional detection with single electrode, the most straightforward design is using different number of strip electrodes for different directions. However, in the case of a large amount of detections, the number of strip electrodes has to increase linearly with the number of directions, resulting in low scalability and great imbalance of device. Therefore, scalable coding configurations which can significantly reduce the number of strip electrodes are highly desirable. The more directions need to be detected, the more obvious gain of the coding configurations. Inspired by the connected structure of spider‐net, two coding configurations of BISNC interfaces are proposed. The first coding configuration is based on electrodes designed with large and small width, i.e., large/small (L/S) coding, to generate output peaks with coding information in terms of relative amplitude. The basic rule of this coding design is to interpret the coding electrodes according to the output peaks with relative large and small amplitude, which is irrelevant of the absolute amplitude of the peaks and can minimize the effect of operation and environment conditions. Thus for each direction, there should be at least one large electrode and one small electrode to create the amplitude variation. Then the second coding configuration is based on the design with and/or without electrode in a predefined position, i.e., 0/1 coding, to generate output peaks with coding information in terms of their positions in time domain. Both coding designs have absolute‐amplitude‐independent detection mechanism, which means that the coding information only relies on the relative amplitude or the time‐domain positions of the output peaks. Thus, the single‐electrode coding designs can offer excellent robustness and reliability in various usage scenarios, e.g., different sliding speeds, sliding forces, and/or ambient humidity, greatly broadening the applications of the BISNC interfaces.

**Figure 1:**
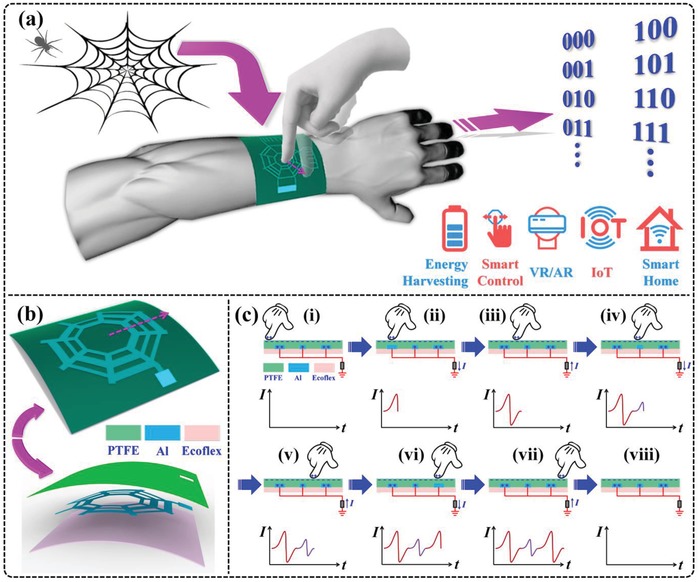
Design configuration of the BISNC interface and operation principle. a) Schematic diagram of the spider‐net‐inspired BISNC interface and its diversified applications. b) Schematic diagram and layer structure of the BISNC interface with L/S coding. c) Operation principle of the L/S coding design and the corresponding output signals.

The schematic diagram of a BISNC interface attached on a human arm is illustrated in **Figure** **1**a, indicating that the spider‐net‐inspired interface can be applied for a wide range of applications, such as energy harvesting, smart control, VR/AR, Internet of Things (IoT), smart home/building, etc. Figure 1b shows the schematic of a BISNC interface with L/S coding, with all the electrodes connecting together into one single electrode. The device consists of only three thin layers, i.e., Ecoflex substrate, patterned Al electrode, and polytetrafluoroethylene (PTFE) friction layer, resulting in a very succinct device configuration. With the L/S coding design, large output peak is generated when sliding across electrode with large width, and similarly, small output peak is generated when sliding across electrode with small width. Then the output peaks with relative large and small amplitude can be adopted to interpret the coding information on the electrodes.

The basic operation principle of the single‐electrode BISNC interface with L/S coding can be explained using the schematics shown in Figure 1c, where finger is sliding on the interface with relatively constant speed. Electrode pattern with large‐small‐large electrode width is used as an example here (the pattern indicated by the dash arrow in Figure 1b). First, when finger wearing nitrile glove comes into contact with the friction surface of the device, i.e., PTFE, electrons are injected from the nitrile glove surface to the PTFE surface due to their different electron affinities. Thereby, nitrile glove surface contains net positive charges while PTFE surface ends up with net negative charges. Accordingly, the three strip electrodes are coupled with different amounts of positive charges by the negative PTFE surface, with large amount of charges on the electrode with large width and small amount of charges on the electrode with small width (Figure 1c‐i). The moment finger slides on the area of the first strip electrode (with large width) from the center area, a large amount of positive charges on that electrode are forced to flow into ground due to the arisen of electric potential difference, resulting in a large positive peak in the external circuit (Figure 1c‐ii). Next, when finger slides out of the area of the first strip electrode, a large amount of positive charges are forced to flow back to the electrode. Then a large negative peak is generated in the external circuit (Figure 1c‐iii). Then when finger slides on the second strip electrode (with small width), a small amount of positive charges on that electrode are forced to flow into ground, generating a small positive peak in the external circuit, due to the small electrode width (Figure 1c‐iv). The moment when finger slides out of the second electrode, a small amount of positive charges are forced to flow back to the electrode and a small negative peak is generated in the external circuit (Figure 1c‐v). Similarly, when finger slides on and out of the third strip electrode (with large width), a large amount of positive charges are forced to flow between that electrode and ground, generating a large positive peak and a large negative peak in the external circuit (Figure 1c‐vi,vii). Then when finger leaves the device surface, no output peak is generated (Figure 1c‐viii). Therefore, when finger slides across electrodes with large and small widths, output signals with different amplitudes are generated in the external circuit. In other words, the coding electrode pattern in the sliding direction can be decoded accordingly based on the relative amplitude of the output peaks.

**Figure 2:**
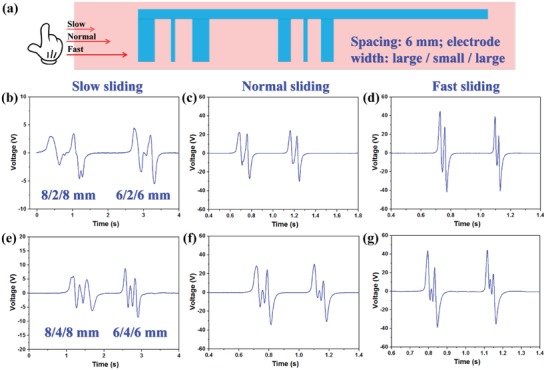
Optimization characteristics of electrode width. a) Schematic of the electrode patterns for the measurement. The generated output peaks from the 8/2/8 and 6/2/6 mm electrode patterns under finger sliding in a b) slow, c) normal, and d) fast manner. The generated output peaks from the 8/4/8 and 6/4/6 mm electrode patterns under finger sliding in a e) slow, f) normal, and g) fast manner.

To determine the suitable electrode width and spacing, measurements with both electrode width and spacing varying from 6 to 2 mm are conducted, as shown in Figure S1 (Supplementary Information). According to the experimental results, it is concluded that certain electrode width and spacing are required for the finger sliding application. Normally speaking, electrode spacing of 6 mm can achieve clear signal identification with separated output peaks. Then to decide the electrode width for large and small electrodes, measurements as shown in **Figure** **2** are conducted. The schematic in Figure 2a indicates that two sets of large/small/large electrode patterns with different dimensions are connected together and investigated under three sliding speeds (slow ≈38 mm s^−1^, normal ≈97 mm s^−1^, and fast ≈152 mm s^−1^). The electrode spacing in both patterns is kept as constant of 6 mm. The measurements in Figure 2b–d are conducted with electrode width of the large/small/large patterns of 8/2/8 mm and 6/2/6 mm, under slow, normal, and fast sliding, respectively. Similarly, the measurements in Figure 2e–g are conducted with electrode width of 8/4/8 mm and 6/4/6 mm. From the results, it can be observed that with larger difference in the electrode width, the variation of the output signal amplitude is more obvious. But if the electrode with small width is too narrow, its generated output peak will have limited amplitude due to the small contact area, which is easily submerged in the large output peaks from the electrodes with large width. In this regard, electrode widths of 8 and 4 mm are adopted for the electrodes in the L/S coding configuration.

**Figure 3:**
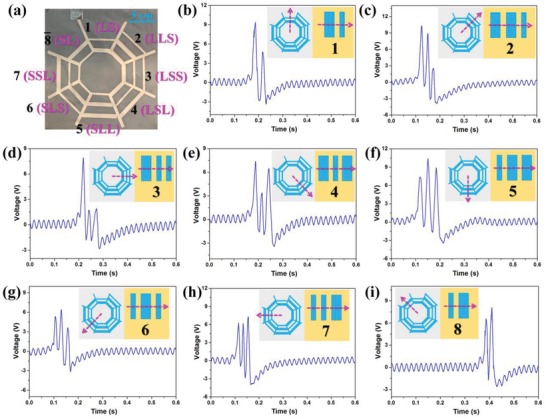
The eight‐direction BISNC interface with L/S coding and wider spacing (electrode width of 8 and 4 mm, spacing of 15 mm). a) The photograph of the device. b–i) The corresponding output peaks when finger sliding across the eight electrode patterns. Insets show the sliding directions and the electrode patterns.

Based on the previous optimization results, a BISNC interface is first fabricated with large electrode width of 8 mm, small electrode width of 4 mm, and electrode spacing of 6 mm. In the ideal case, electrode with larger width should produce higher output peak due to the larger contact area during sliding. However, due to the relatively large size of human finger of ≈15 mm and the small electrode spacing of 6 mm, finger may cover two adjacent electrodes simultaneously and cause the overlapping of generated output peaks, as shown in Figure S2 (Supplementary Information). The negative peak of the former signal may overlap with the positive peak of the latter signal, leading to amplitude reduction of the second signal. Thus, amplitude of the output peaks may not come out as expected—large amplitude from large electrode and small amplitude from small electrode. In order to accurately recognize the electrode patterns, a forward/backward sliding strategy is proposed together with a signal interpretation table, as illustrated in Figure S2 and Table S1 (Supplementary Information). Then to minimize the negative effect of overlapping signals and develop a more intuitive detection mechanism, another eight‐direction BISNC interface with larger electrode spacing (15 mm) is fabricated and investigated, with its photograph shown in **Figure** **3**a. Benefited from the design of larger electrode spacing, the overlapping of output signals can be minimized and thereby output signal pattern corresponding to the electrode pattern can be achieved, i.e., large amplitude from large electrode. The generated output signals when sliding across the eight directions are illustrated in Figure 3b–i, respectively. According to the measurement results, it can be observed that the amplitude variation of the output signals is able to follow the width variation of the sensing electrodes correspondingly. Furthermore, no backward sliding is required in order to determine the coding information in electrodes. In this way, the interpretation mechanism in the processing circuit can then be significantly simplified.

The triboelectric mechanism enables the BISNC interface to also function as an energy harvester to scavenge mechanical energy from various contact and sliding motions such as human tapping. The measurement results from human hand tapping are summarized in Figure S3 (Supplementary Information), where the recorded output voltage (on a 100 MΩ load), charge, and short circuit current of the device are 385 V, 482 nC, and 6.25 µA, respectively. Compared to outputs with finger sliding, the outputs from hand tapping are greatly improved due to the much larger contact area of human palm and the device, inducing more charges flowing in the external circuit with each tapping motion. When changing the resistance of the connected external loads, the BISNC interface can produce a maximum output power of 3.2 mW on a matched resistance of 13 MΩ (with tapping frequency of ≈3.5 Hz). Therefore, the BISNC interface not only can be used as self‐powered interface, but also can be used as energy harvester/generator to scavenge energy from human motions and store in capacitor for the potential operation of the back‐end circuits, toward the eventual realization of self‐powered and battery‐less functional interfaces.

## 3 Advanced BISNC Interface as Three‐Bit Binary Code Interface and Application

**Figure 4:**
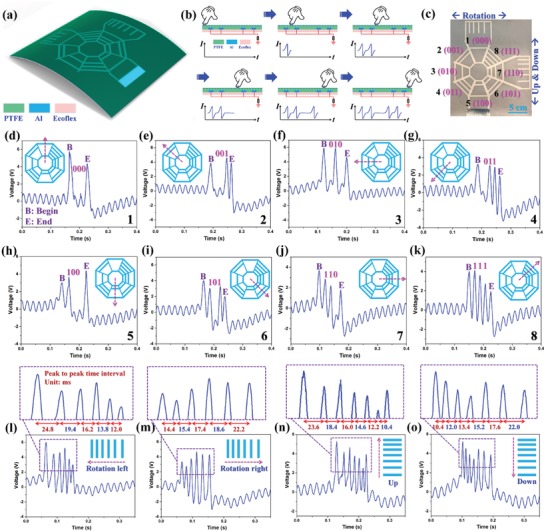
Advanced BISNC interface with 0/1 coding (electrode width of 4 mm, spacing of 6 mm). a) Schematic diagram of the eight‐direction BISNC interface with 0/1 coding. b) The operation principle of 0/1 coding design and the corresponding output signals. c) The photograph of the device. d–k) The corresponding output peaks when finger sliding across the eight electrode patterns. Insets show the eight different sliding directions, and “B” and “E” denote the output peaks from the beginning and ending reference electrode. The generated signals from the top electrode pattern with finger sliding to the l) left and m) right direction. The generated signals from the right electrode pattern with finger sliding to the n) up and o) down direction.

Although intuitive signal patterns can be achieved using L/S coding with larger electrode spacing, the comparison and judgement of signal amplitude of large, small, and equivalent are not so straightforward in the processing circuit. Besides, the larger electrode spacing greatly increases the device dimension. Meanwhile, another information coding scheme, i.e., 0/1 coding, has the detection mechanism irrelevant of the signal amplitude, but only related to signal positions in time domain. Thus, even with some overlapping of signals due to the small spacing, the coding information still can be correctly decoded as long as the generated signal positions are distinguishable. Herein, another advanced eight‐direction BISNC interface with 0/1 coding is proposed, with the electrode width of 4 mm and electrode spacing of only 6 mm. As depicted in **Figure** **4**a, all the electrode patterns are equipped with one beginning electrode and one ending electrode as reference points for the recognition of output peaks' positions in time domain. Then the middle three electrodes are coded with three‐bit binary code of 000, 001, 010, 011, 100, 101, 110, and 111 (where “1” means that there is one strip electrode in that position and “0” meaning that there is no strip electrode in that position). Hence, when sliding across the beginning electrode, there is always one output peak generated. Then if there is a strip electrode in the first coding position, another output peak is generated. If there is no strip electrode in the first coding position, no output peak is generated and signal remains as 0. Similarly, same for the second and the third coding positions. After three coding electrodes, another output peak is generated from the ending electrode as another reference point. Therefore, based on the information‐coding electrode patterns, output signals with different peak positions in time domain will be generated, which can be decoded to reveal the coded information. The amplitude of output peaks can be ignored in this decoding process, offering a more straightforward and reliable detection mechanism.

The detail operation principle of electrode pattern “101” as an example is illustrated in Figure 4b. When finger slides across the beginning strip electrode, an output peak is generated in the external circuit. Then when finger slides across the first coded strip electrode (1), another output peak is generated. Moving forward, when finger is sliding on the area of the second coded strip electrodes (0), no output peak is generated since no strip electrode is there due to the “0” coding. After that, two output peaks are generated when finger slides across the third coded strip electrode (1) and the ending strip electrode. Since finger is sliding on the interface with a relatively constant speed, the 0/1 coding information can be interpreted from the positions of the generated peaks in the time domain.

Figure 4c shows the photograph of the fabricated eight‐direction BISNC interface, with two additional sensing patterns for potential up/down and rotation control. The corresponding output signals for these eight directions are presented in Figure 4d–k. The letter “B” and “E” in the graphs denote the beginning reference point and ending reference point from the two reference electrodes. With the help of these two reference points, the coding information in the electrode patterns can be interpreted accordingly. For example, if there is no output peak between the two reference points, it means that the coding information is 000. If there are three output peaks between the two reference points, it means that the coding information is 111. With one output peak in between, the coding information can be 001, 010, or 100, according to the position of the peak, i.e., closer to the ending reference point (001), in the middle of the two reference points (010), or closer to the beginning reference point (100). Similarly, if there are two output peaks in between, the coding information can be 011, 101, or 110, according to the positions of the two output peaks, i.e., both closer to the ending reference point (011), one closer to the beginning reference point and one closer to the ending reference point (101), or both closer to the beginning reference point (110). Through measuring the peak position with respect to the two reference points in time domain, the coding information in each direction can be decoded. The robustness of the device and detection mechanism regarding to the variation of sliding speed is discussed in Figure S4 (Supplementary Information), indicating that as long as the variation of sliding speed is within 40%, the coding information can be correctly decoded and all directions can be recognized.

The BISNC interface with 0/1 coding can be potentially adopted for 3D drone control, with the eight 0/1 coding electrode patterns for multidirectional in‐plane control and the two additional electrode patterns for rotation and up/down control. In this regard, 3D drone control can be realized using the BISNC interface with only one electrode output. To differentiate the output signal patterns from those of in‐plane control, electrode pattern for rotation control is configured with six strip electrodes while electrode pattern for up/down control is configured with seven strip electrodes. To further distinguish the direction of sliding left/right and up/down, the spacing of these electrodes is varied from small to large in each pattern. Then according to the number of output peaks and the trend of the peak intervals (increasing or decreasing), left/right rotation control and up/down control can be determined. Figure 4l,m shows the generated signals for the rotation control and the enlarged view of the output peaks and their time intervals. When sliding left, there are six peaks in the generated signal and their time intervals are decreasing. Then when sliding right, their time intervals are increasing. Figure 4n,o shows the generated signals for the up/down control and enlarged view of the output peaks and their time intervals. When sliding up, there are seven peaks in the generated signal and their time intervals are decreasing. Then when sliding down, their time intervals are increasing. The trend of the time intervals in rotation control and up/down control is illustrated in Figure S5 (Supplementary Information).

**Figure 5:**
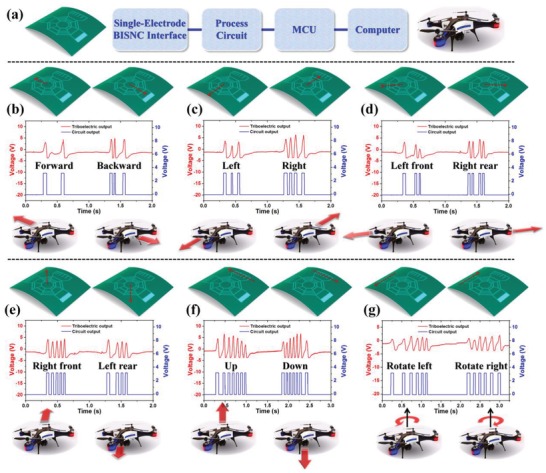
Demonstration of 3D drone control using the BISNC interface. a) Block diagram of the entire control system. The generated triboelectric output from the BISNC interface and the output after the processing circuit for the drone movement control in b) forward/backward direction, c) left/right direction, d) left front/right rear direction, e) right front/left rear direction, f) up/down direction, and g) left/right rotation. The insets show the sliding operations on the BISNC interface and the corresponding drone movements.

Due to the excellent robustness and reliability of the 0/1‐coding eight‐direction BISNC interface, it is adopted for 3D control of virtual drone in cyber space to show its practical usability. As shown in **Figure** **5**a, the entire control system is composed of the eight‐direction BISNC interface as the control interface, a processing circuit, a microcontroller unit (MCU) for signal recognition and control command generation, and a computer for drone control. The processing circuit mainly consists of a comparator circuit to convert the triboelectric peaks into square wave pulses for easy recognition by the MCU. Through programming the MCU, the BISNC interface can be adopted to control the 3D motions of the drone intuitively according to different finger sliding operations. First, the eight directions with 0/1 coding electrodes are programed to control the in‐plane motions of the drone, i.e., forward, backward, left, right, left front direction, right rear direction, right front direction, and left rear direction. The generated triboelectric signals from the BISNC interface and the square wave signals after the processing circuit for the eight directions are depicted in Figure 5b–e. The corresponding finger sliding traces on the BISNC interface and drone motions in 3D space are also shown as insets. Then the right electrode pattern of seven strip electrodes with varying spacing is programed to control the up/down motion of the drone when sliding up or down by finger. Besides, the top electrode pattern of six strip electrodes with varying spacing is programed to control the in‐plane rotation motion of the drone under left/right finger sliding. The generated triboelectric signals and the square wave signals for the up/down and in‐plane rotation drone control are illustrated in Figure 5f,g, together with the finger sliding traces and drone motions. The real‐time control of the virtual drone in 3D space can be found in Video S1 (Supplementary Information).

## 4 Scalability and Diversified Applications of BISNC Interface

**Figure 6:**
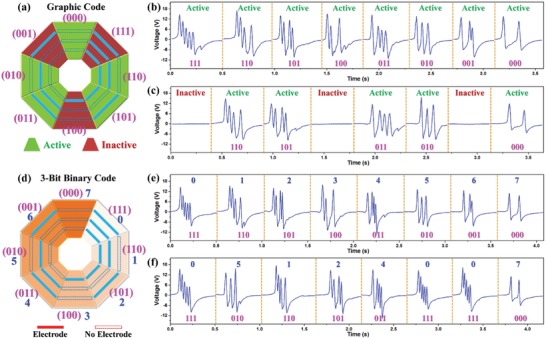
Security code interface using the eight‐direction 0/1‐coding BISNC interface. a) Graphic code system. b) Graphic code with all the eight directions set as active. c) Graphic code with directions of east, southeast, southwest, west, and north set as active. d) Digital code system using three‐bit binary code. e) Digital code with “01234567” coding information. f) Digital code with “05124007” coding information.

Except for the application in 3D control, the spider‐net‐inspired BISNC interfaces exhibit outstanding scalability for other applications such as security code, just like the spider‐net that can be woven wider and wider. For instance, four directions can be defined using two‐bit coding electrodes, eight directions can be defined using three‐bit coding electrodes, 16 directions can be defined using four‐bit coding electrodes, etc. First, the eight‐direction 0/1‐coding BISNC interface is adopted as the interface of security code system. Two types of coding strategies are proposed using the device, i.e., graphic code and digital code, as depicted in **Figure** **6**a,d. In the graphic code system, each direction is only regarded as active or inactive. To set the code, one can select which direction is active and which direction is inactive. Then in order to trigger the code, one only needs to remember all the active directions and slide, regardless of the sliding order. Thus, the graphic code is very straightforward and easy to remember, fast response in both triggering and recognition, and more importantly, no specific order to slide. As long as one slides all the “set” active directions, the code is considered as correct. Figure 6b,c shows the example of the graphic code with all the directions set as active and only directions of east, southeast, southwest, west, and north set as active, respectively. In the digital code system, each direction is treated as one digit of octal code, i.e., from 0 to 7. To set the code, one is required to slide each direction in a specific order. Then to trigger the code, one needs to slide all the selected directions in the same order. Thus to successfully enter the code, one needs to remember both the numbers (directions) and the order. It can be seen that the digital code system is more similar to the traditional code system and it offers higher safety level compared to the graphic code. Figure 6e,f shows the example of the digital code with “01234567” coding information and “05124007” coding information, respectively. In practical applications, the BISNC interface can be configured into one of the above two code systems according to the usage requirements and user's preference.

**Figure 7:**
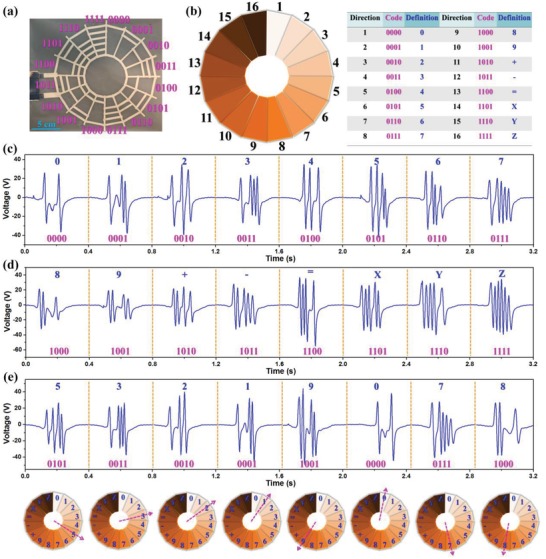
Sixteen‐direction 0/1‐coding BISNC interface. a) Photograph and coding of the device. b) Schematic diagram of the device and an example of BCD. c,d) The output signals generated when sliding across the 16 directions. e) Illustration of using the device for decimal security code with step‐by‐step sliding indication.

To demonstrate the excellent scalability of the spider‐net‐inspired coding interfaces, 16‐direction 0/1‐coding BISNC interface with four‐bit binary code is shown in **Figure** **7**a. Figure 7c,d depicts the corresponding output signals from the 16 directions, showing the four‐bit binary coded electrodes between the two reference electrodes can be differentiated according to the time‐domain output peaks. Except for the security code system, the 16‐direction interface can also be used for Binary Coded Decimal (BCD) and other functions,^88^ as illustrated in Figure 7b. For instance, the 16‐direction interface can be used to set the spatial coordinates of a point (15, 35, 22) by consecutively sliding across direction 14, 13, 2, 6, 15, 13, 4, 6, 16, 13, 3, and 3 (“X” “=” “1” “5” “Y” “=” “3” “5” “Z” “=” “2” “2”). Another example is that it can function as the input interface of a calculator, e.g., calculate the result of “−138 − 56 + 31 =” by consecutively sliding across direction 12, 2, 4, 9, 12, 6, 7, 11, 4, 2, and 13 (“−” “1” “3” “8” “−” “5” “6” “+” “3” “1” “=”), as depicted in Figure S6 (Supplementary Information). Then the calculator will output the correct result. Besides, the binary code system can also be adopted for potential applications in classification method,^89^ gray code to perform error correction in communication,^90^ etc. When using in a security code system, the 16‐direction BISNC interface can offer each digit of the password varying within the decimal number (i.e., from 0 to 9). Figure 7e shows the corresponding output signals for the coding information of “53219078,” together with step‐by‐step sliding indications on the respective directions.

**Figure 8:**
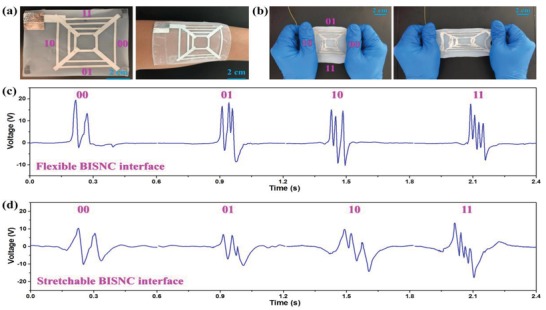
Toward flexible wearable applications. a) A flexible four‐direction BISNC interface. b) A stretchable four‐direction BISNC interface. c) The generated signals from the flexible BISNC interface. d) The generated signals from the stretchable BISNC interface.

In addition, the highly scalable BISNC interfaces can be equipped with flexible and/or stretchable compatibility toward flexible wearable applications. A flexible four‐direction BISNC interface and a stretchable four‐direction BISNC interface are developed and shown in **Figure** **8**a,b, respectively, indicating great wearable compatibility of both devices. The flexible BISNC interface is based on the same device structure and materials as the previous BISNC interfaces, while the stretchable BISNC interface is fabricated with encapsulated liquid metal inside of Ecoflex channels, forming the coding electrode patterns. The detail fabrication process of the stretchable BISNC interface is illustrated in Figure S7 (Supplementary Information). The output signals from the flexible and stretchable BISNC interfaces are depicted in Figure 8c,d, respectively. Video S2 and Video S3 in the Supplementary Information present the finger sliding operation of both devices and the corresponding output signals. From the results, it can be observed that the coding information along the four directions can be clearly interpreted from the generated peaks for both devices. According to the above demonstrations, the concept of single‐electrode BISNC interface exhibits outstanding scalability, showing great potentials in diverse control, security, flexible and wearable applications.

## 5 Conclusions

In summary, highly scalable and single‐electrode BISNC interfaces are developed through connecting the information‐coding electrodes into the layout of spider‐net structure. Compared to the previously reported triboelectric interfaces, the developed BISNC interfaces exhibit superior advantages of single‐electrode output for multidirectional 2D/3D control, high scalability, excellent robustness, and reliability in various usage scenarios. Because the signal detection mechanism is independent of the absolute amplitude of output signals and thereby immunity to different sliding force, sliding speed, ambient humidity, etc. Besides, the developed BISNC interfaces can also be used as energy harvester to scavenge the hand tapping energy with good performance. In addition, real‐time drone control in 3D space using the BISNC interface is successfully demonstrated. Moreover, different scalable interfaces have been developed for security code system, BCD, and flexible wearable applications. The highly scalable and single‐electrode BISNC interfaces enabled by the spider‐net‐coding design have great potentials in diverse usage scenarios, including 2D/3D control, VR/AR, security code system, wearable electronics, robotics, smart home/factory/building, IoT, etc.

## 6 Experimental Section

*Fabrication of the BISNC Interface*: PTFE thin film was first cut into the size of the designed interface. Then aluminum tape electrodes were attached on the backside of the PTFE thin film, forming the structure layout of connected spider‐net. All the electrodes are connected together, and thus there is only one sensing electrode for the BISNC interface. After that, Ecoflex 00–30 solution with mass ratio of 1:1 (Part A:Part B) was mixed thoroughly and coated on top of the electrode as insulation and protection layer. Last, the BISNC interface was cured overnight at room temperature.

*Fabrication of the Stretchable BISNC Interface*: The process was started from molding substrate preparation and cleaning. Next, foam tapes were attached on the substrate forming the fluidic channels. Then Ecoflex 00–30 solution was casting on top and cured at room temperature. Next, Ecoflex with fluidic channels was peeled off from the substrate, and then liquid metal filling into the fluidic channels was conducted using syringe. Last, another Ecoflex layer was casted on top and cured for encapsulation.

*Characterization of the BISNC Interface*: The output voltage of the BISNC interface was measured by DSO‐X3034A oscilloscope (Agilent) with a high impedance probe of 100 MΩ. The output current and charge were measured by Keithley 6514 Electrometer. Programmable Arduino UNO was adopted as the microcontroller unit for signal recognition and command generation.

*Study Participation*: Prior to participation in the experiments, informed consent was obtained from the volunteer in all experiments.

## Conflict of Interest

The authors declare no conflict of interest.

## Supporting information

Supplementary

Supplementary

Supplementary

Supplementary

## References

[1] M. Stoppa , A. Chiolerio , Sensors 2014, 14, 11957. 25004153 10.3390/s140711957 PMC4168435

[2] X. Wang , L. Dong , H. Zhang , R. Yu , C. Pan , Z. L. Wang , Adv. Sci. 2015, 2, 1500169. 10.1002/advs.201500169 PMC5115318 27980911

[3] H.‐J. Yoon , H. Ryu , S.‐W. Kim , Nano Energy 2018, 51, 270.

[4] H. Chen , Y. Song , X. Cheng , H. Zhang , Nano Energy 2019, 56, 252.

[5] S. Wang , J. Xu , W. Wang , G.‐J. N. Wang , R. Rastak , F. Molina‐Lopez , J. W. Chung , S. Niu , V. R. Feig , J. Lopez , Nature 2018, 555, 83. 29466334 10.1038/nature25494

[6] M. L. Hammock , A. Chortos , B. C. K. Tee , J. B. H. Tok , Z. Bao , Adv. Mater. 2013, 25, 5997. 24151185 10.1002/adma.201302240

[7] A. Chortos , J. Liu , Z. Bao , Nat. Mater. 2016, 15, 937. 27376685 10.1038/nmat4671

[8] H. Jinno , K. Fukuda , X. Xu , S. Park , Y. Suzuki , M. Koizumi , T. Yokota , I. Osaka , K. Takimiya , T. Someya , Nat. Energy 2017, 2, 780.

[9] J. Chen , Y. Huang , N. Zhang , H. Zou , R. Liu , C. Tao , X. Fan , Z. L. Wang , Nat. Energy 2016, 1, 16138.

[10] S. J. Kim , H. E. Lee , H. Choi , Y. Kim , J. H. We , J. S. Shin , K. J. Lee , B. J. Cho , ACS Nano 2016, 10, 10851. 28024371 10.1021/acsnano.6b05004

[11] S. Z. Guo , K. Qiu , F. Meng , S. H. Park , M. C. McAlpine , Adv. Mater. 2017, 29, 1701218. 10.1002/adma.201701218 PMC5509487 28474793

[12] S. Lee , A. Reuveny , J. Reeder , S. Lee , H. Jin , Q. Liu , T. Yokota , T. Sekitani , T. Isoyama , Y. Abe , Nat. Nanotechnol. 2016, 11, 472. 26809055 10.1038/nnano.2015.324

[13] C. Wang , X. Li , H. Hu , L. Zhang , Z. Huang , M. Lin , Z. Zhang , Z. Yin , B. Huang , H. Gong , Nat. Biomed. Eng. 2018, 2, 687. 30906648 10.1038/s41551-018-0287-x PMC6428206

[14] W. Gao , S. Emaminejad , H. Y. Y. Nyein , S. Challa , K. Chen , A. Peck , H. M. Fahad , H. Ota , H. Shiraki , D. Kiriya , Nature 2016, 529, 509. 26819044 10.1038/nature16521 PMC4996079

[15] M. Bariya , H. Y. Y. Nyein , A. Javey , Nat. Electron. 2018, 1, 160.

[16] B. C.‐K. Tee , A. Chortos , A. Berndt , A. K. Nguyen , A. Tom , A. McGuire , Z. C. Lin , K. Tien , W.‐G. Bae , H. Wang , Science 2015, 350, 313. 26472906 10.1126/science.aaa9306

[17] K.‐I. Jang , K. Li , H. U. Chung , S. Xu , H. N. Jung , Y. Yang , J. W. Kwak , H. H. Jung , J. Song , C. Yang , Nat. Commun. 2017, 8, 15894. 28635956 10.1038/ncomms15894 PMC5482057

[18] F. Zhang , Y. Zang , D. Huang , C.‐A. Di , D. Zhu , Nat. Commun. 2015, 6, 8356. 26387591 10.1038/ncomms9356 PMC4595753

[19] Y. C. Lai , J. Deng , R. Liu , Y. C. Hsiao , S. L. Zhang , W. Peng , H. M. Wu , X. Wang , Z. L. Wang , Adv. Mater. 2018, 30, 1801114. 10.1002/adma.201801114 29869431

[20] Y. Liu , M. Pharr , G. A. Salvatore , ACS Nano 2017, 11, 9614. 28901746 10.1021/acsnano.7b04898

[21] H. Fang , K. J. Yu , C. Gloschat , Z. Yang , E. Song , C.‐H. Chiang , J. Zhao , S. M. Won , S. Xu , M. Trumpis , Nat. Biomed. Eng. 2017, 1, 0038. 28804678 10.1038/s41551-017-0038 PMC5552067

[22] S. Park , S. W. Heo , W. Lee , D. Inoue , Z. Jiang , K. Yu , H. Jinno , D. Hashizume , M. Sekino , T. Yokota , Nature 2018, 561, 516. 30258137 10.1038/s41586-018-0536-x

[23] C.‐C. Kim , H.‐H. Lee , K. H. Oh , J.‐Y. Sun , Science 2016, 353, 682. 27516597 10.1126/science.aaf8810

[24] R. Cao , X. Pu , X. Du , W. Yang , J. Wang , H. Guo , S. Zhao , Z. Yuan , C. Zhang , C. Li , ACS Nano 2018, 12, 5190. 29771494 10.1021/acsnano.8b02477

[25] H. Liu , C. Hou , J. Lin , Y. Li , Q. Shi , T. Chen , L. Sun , C. Lee , Appl. Phys. Lett. 2018, 113, 203901.

[26] R. K. Gupta , Q. Shi , L. Dhakar , T. Wang , C. H. Heng , C. Lee , Sci. Rep. 2017, 7, 41396. 28120924 10.1038/srep41396 PMC5264648

[27] G. T. Hwang , H. Park , J. H. Lee , S. Oh , K. I. Park , M. Byun , H. Park , G. Ahn , C. K. Jeong , K. No , Adv. Mater. 2014, 26, 4880. 24740465 10.1002/adma.201400562

[28] Q. Shi , T. Wang , T. Kobayashi , C. Lee , Appl. Phys. Lett. 2016, 108, 193902.

[29] C. Dagdeviren , B. D. Yang , Y. Su , P. L. Tran , P. Joe , E. Anderson , J. Xia , V. Doraiswamy , B. Dehdashti , X. Feng , B. Lu , R. Poston , Z. Khalpey , R. Ghaffari , Y. Huang , M. J. Slepian , J. A. Rogers , Proc. Natl. Acad. Sci. U. S. A. 2014, 111, 1927. 24449853 10.1073/pnas.1317233111 PMC3918766

[30] Q. Shi , H. Wu , H. Wang , H. Wu , C. Lee , Adv. Energy Mater. 2017, 7, 1701300.

[31] K. Dong , Z. Wu , J. Deng , A. C. Wang , H. Zou , C. Chen , D. Hu , B. Gu , B. Sun , Z. L. Wang , Adv. Mater. 2018, 30, 1804944. 10.1002/adma.201804944 30256476

[32] H. Guo , X. Pu , J. Chen , Y. Meng , M.‐H. Yeh , G. Liu , Q. Tang , B. Chen , D. Liu , S. Qi , Sci. Rob. 2018, 3, eaat2516. 10.1126/scirobotics.aat2516 33141730

[33] F.‐R. Fan , Z.‐Q. Tian , Z. L. Wang , Nano Energy 2012, 1, 328.

[34] B. Shi , Z. Li , Y. Fan , Adv. Mater. 2018, 30, 1801511. 10.1002/adma.201801511 30043422

[35] T. Chen , Q. Shi , M. Zhu , T. He , L. Sun , L. Yang , C. Lee , ACS Nano 2018, 12, 11561. 30335958 10.1021/acsnano.8b06747

[36] B. Chen , Y. Yang , Z. L. Wang , Adv. Energy Mater. 2018, 8, 1702649.

[37] C. Wu , A. C. Wang , W. Ding , H. Guo , Z. L. Wang , Adv. Energy Mater. 2018, 8, 1802906.

[38] D. Li , W. Y. Lai , Y. Z. Zhang , W. Huang , Adv. Mater. 2018, 30, 1704738. 10.1002/adma.201704738 29319214

[39] L. Chen , Q. Shi , Y. Sun , T. Nguyen , C. Lee , S. Soh , Adv. Mater. 2018, 30, 1802405. 10.1002/adma.201802405 30129287

[40] W. Wu , X. Cao , J. Zou , Y. Ma , X. Wu , C. Sun , M. Li , N. Wang , Z. Wang , L. Zhang , Adv. Funct. Mater. 2018, 1806331.

[41] J. Sun , X. Pu , M. Liu , A. Yu , C. Du , J. Zhai , W. Hu , Z. L. Wang , ACS Nano 2018, 12, 6147. 29851468 10.1021/acsnano.8b02479

[42] M. Zhu , Q. Shi , T. He , Z. Yi , Y. Ma , B. Yang , T. Chen , C. Lee , ACS Nano 2019, 13, 1940. 30741521 10.1021/acsnano.8b08329

[43] C. Sun , Q. Shi , D. Hasan , M. S. Yazici , M. Zhu , Y. Ma , B. Dong , Y. Liu , C. Lee , Nano Energy 2019, 58, 612.

[44] Y.‐C. Lai , Y.‐C. Hsiao , H.‐M. Wu , Z. L. Wang , Adv. Sci. 2019, 6, 1801883. 10.1002/advs.201801883 PMC6402409 30886807

[45] J. Chung , H. Yong , H. Moon , Q. V. Duong , S. T. Choi , D. Kim , S. Lee , Adv. Sci. 2018, 5, 1801054. 10.1002/advs.201801054 PMC6247056 30479934

[46] Y. Ji , K. Zhang , Y. Yang , Adv. Sci. 2018, 5, 1700622. 10.1002/advs.201700622 PMC5826984 29619310

[47] S. Y. Kuang , G. Zhu , Z. L. Wang , Adv. Sci. 2018, 5, 1700658. 10.1002/advs.201700658 PMC5826983 29619315

[48] H. Ryu , J. H. Lee , U. Khan , S. S. Kwak , R. Hinchet , S.‐W. Kim , Energy Environ. Sci. 2018, 11, 2057.

[49] Z. Liu , Y. Ma , H. Ouyang , B. Shi , N. Li , D. Jiang , F. Xie , D. Qu , Y. Zou , Y. Huang , H. Li , C. Zhao , P. Tan , M. Yu , Y. Fan , H. Zhang , Z. L. Wang , Z. Li , Adv. Funct. Mater. 2019, 29, 1807560.

[50] Q. Shi , T. He , C. Lee , Nano Energy 2019, 57, 851.

[51] X. Li , G. Xu , X. Xia , J. Fu , L. Huang , Y. Zi , Nano Energy 2019, 56, 40.

[52] Y. Jie , X. Jia , J. Zou , Y. Chen , N. Wang , Z. L. Wang , X. Cao , Adv. Energy Mater. 2018, 8, 1703133.

[53] J. Chen , X. Pu , H. Guo , Q. Tang , L. Feng , X. Wang , C. Hu , Nano Energy 2018, 43, 253.

[54] Q. Jiang , C. Wu , Z. Wang , A. C. Wang , J.‐H. He , Z. L. Wang , H. N. Alshareef , Nano Energy 2018, 45, 266.

[55] S. Jin , Y. Wang , M. Motlag , S. Gao , J. Xu , Q. Nian , W. Wu , G. J. Cheng , Adv. Mater. 2018, 30, 1705840. 10.1002/adma.201705840 29356129

[56] Y. Hu , Z. Zheng , Nano Energy 2019, 56, 16.

[57] L. Gao , D. Hu , M. Qi , J. Gong , H. Zhou , X. Chen , J. Chen , J. Cai , L. Wu , N. Hu , Nanoscale 2018, 10, 19781. 30328888 10.1039/c8nr05957h

[58] J. Nie , Z. Ren , J. Shao , C. Deng , L. Xu , X. Chen , M. Li , Z. L. Wang , ACS Nano 2018, 12, 1491. 29341585 10.1021/acsnano.7b08014

[59] G. Zhao , J. Yang , J. Chen , G. Zhu , Z. Jiang , X. Liu , G. Niu , Z. L. Wang , B. Zhang , Adv. Mater. Technol. 2019, 4, 1800167.

[60] T. Chen , M. Zhao , Q. Shi , Z. Yang , H. Liu , L. Sun , J. Ouyang , C. Lee , Nano Energy 2018, 51, 162.

[61] T. Chen , Q. Shi , Z. Yang , J. Liu , H. Liu , L. Sun , C. Lee , Nanomaterials 2018, 8, 503. 10.3390/nano8070503 PMC6071088 29986476

[62] H. Wu , Q. Shi , F. Wang , A. V. Y. Thean , C. Lee , Small Methods 2018, 2, 1800078.

[63] W. Seung , M. K. Gupta , K. Y. Lee , K.‐S. Shin , J.‐H. Lee , T. Y. Kim , S. Kim , J. Lin , J. H. Kim , S.‐W. Kim , ACS Nano 2015, 9, 3501. 25670211 10.1021/nn507221f

[64] X. Wang , Y. Zhang , X. Zhang , Z. Huo , X. Li , M. Que , Z. Peng , H. Wang , C. Pan , Adv. Mater. 2018, 30, 1706738. 10.1002/adma.201706738 29411908

[65] L. Zheng , Y. Wu , X. Chen , A. Yu , L. Xu , Y. Liu , H. Li , Z. L. Wang , Adv. Funct. Mater. 2017, 27, 1606408.

[66] X.‐S. Zhang , M.‐D. Han , R.‐X. Wang , B. Meng , F.‐Y. Zhu , X.‐M. Sun , W. Hu , W. Wang , Z.‐H. Li , H.‐X. Zhang , Nano Energy 2014, 4, 123.

[67] X.‐S. Zhang , J. Brugger , B. Kim , Nano Energy 2016, 20, 37.

[68] S. Lee , H. Wang , Q. Shi , L. Dhakar , J. Wang , N. V. Thakor , S.‐C. Yen , C. Lee , Nano Energy 2017, 33, 1.

[69] X. Chen , X. Pu , T. Jiang , A. Yu , L. Xu , Z. L. Wang , Adv. Funct. Mater. 2017, 27, 1603788.

[70] C. B. Han , C. Zhang , X. H. Li , L. Zhang , T. Zhou , W. Hu , Z. L. Wang , Nano Energy 2014, 9, 325.

[71] R. Pan , W. Xuan , J. Chen , S. Dong , H. Jin , X. Wang , H. Li , J. Luo , Nano Energy 2018, 45, 193.

[72] H. Liu , J. Zhong , C. Lee , S.‐W. Lee , L. Lin , Appl. Phys. Rev. 2018, 5, 041306.

[73] X. Y. Wei , X. Wang , S. Y. Kuang , L. Su , H. Y. Li , Y. Wang , C. Pan , Z. L. Wang , G. Zhu , Adv. Mater. 2016, 28, 6656. 27213998 10.1002/adma.201600604

[74] X. Pu , H. Guo , Q. Tang , J. Chen , L. Feng , G. Liu , X. Wang , Y. Xi , C. Hu , Z. L. Wang , Nano Energy 2018, 54, 453.

[75] Q. Shi , C. Qiu , T. He , F. Wu , M. Zhu , J. A. Dziubanf , R. Walczak , M. R. Yuce , C. Lee , Nano Energy 2019, 60, 545.

[76] M. Chen , X. Li , L. Lin , W. Du , X. Han , J. Zhu , C. Pan , Z. L. Wang , Adv. Funct. Mater. 2014, 24, 5059.

[77] Z. Yuan , X. Du , N. Li , Y. Yin , R. Cao , X. Zhang , S. Zhao , H. Niu , T. Jiang , W. Xu , Z. L. Wang , C. Li , Adv. Sci. 2018, 5, 1700881. 10.1002/advs.201700881 PMC5908373 29721430

[78] K.‐I. Jang , H. U. Chung , S. Xu , C. H. Lee , H. Luan , J. Jeong , H. Cheng , G.‐T. Kim , S. Y. Han , J. W. Lee , Nat. Commun. 2015, 6, 6566. 25782446 10.1038/ncomms7566 PMC4383007

[79] S. Choi , H. Lee , R. Ghaffari , T. Hyeon , D. H. Kim , Adv. Mater. 2016, 28, 4203. 26779680 10.1002/adma.201504150

[80] Y. Liu , K. He , G. Chen , W. R. Leow , X. Chen , Chem. Rev. 2017, 117, 12893. 28991450 10.1021/acs.chemrev.7b00291

[81] S. Wang , J. Y. Oh , J. Xu , H. Tran , Z. Bao , Acc. Chem. Res. 2018, 51, 1033. 29693379 10.1021/acs.accounts.8b00015

[82] E. Romero , V. I. Novoderezhkin , R. van Grondelle , Nature 2017, 543, 355. 28300093 10.1038/nature22012

[83] X. Yang , T. Zhou , T. J. Zwang , G. Hong , Y. Zhao , R. D. Viveros , T.‐M. Fu , T. Gao , C. M. Lieber , Nat. Mater. 2019, 18, 510. 30804509 10.1038/s41563-019-0292-9 PMC6474791

[84] G. Y. Bae , S. W. Pak , D. Kim , G. Lee , D. H. Kim , Y. Chung , K. Cho , Adv. Mater. 2016, 28, 5300. 27159832 10.1002/adma.201600408

[85] Q. Hua , J. Sun , H. Liu , R. Bao , R. Yu , J. Zhai , C. Pan , Z. L. Wang , Nat. Commun. 2018, 9, 244. 29339793 10.1038/s41467-017-02685-9 PMC5770430

[86] H. Wang , Y. Yang , L. Guo , Adv. Energy Mater. 2017, 7, 1601709.

[87] C. Choi , M. K. Choi , S. Liu , M. S. Kim , O. K. Park , C. Im , J. Kim , X. Qin , G. J. Lee , K. W. Cho , Nat. Commun. 2017, 8, 1664. 29162854 10.1038/s41467-017-01824-6 PMC5698290

[88] J. Kim , H. J. Kim , in Int. Conf. Information Science and Applications (Eds: K. Kim , N. Baek ), Springer, Singapore 2018, p. 15.

[89] Z. Chong , W. Cai , C. Yao , in 2018 Joint Int. Advanced Engineering and Technology Research Conf. (JIAET 2018), Atlantis Press, Paris, France 2018, p. 206.

[90] L. Liu , J. Dong , X. Zhang , Opt. Express 2015, 23, 21414. 26367989 10.1364/OE.23.021414

