# Supplementary material for: Self‐Powered Bio‐Inspired Spider‐Net‐Coding Interface Using Single‐Electrode Triboelectric Nanogenerator
Source: Adv Sci (Weinh). 2019 May 29;6(15):1900617. doi: 10.1002/advs.201900617 (PMC6685466; doi:10.1002/advs.201900617)
Supplement: Supplementary file 1 — Supplementary [file ADVS-6-1900617-s002.pdf]

Copyright WILEY-VCH Verlag GmbH & Co. KGaA, 69469 Weinheim, Germany, 2019.

ADVANCED SCIENCE
Open Access

Supporting Information

for *Adv. Sci.,* DOI: 10.1002/advs.201900617

Self-Powered Bio-Inspired Spider-Net-Coding Interface Using Single-Electrode Triboelectric Nanogenerator

*Qiongfeng Shi and Chengkuo Lee**

Supporting Information

Self-Powered Bio-Inspired Spider-Net-Coding Interface Using Single-Electrode Triboelectric Nanogenerator

*Qiongfeng Shi, Chengkuo Lee**

Supporting Information Table

S1. Optimization of electrode width and spacing…………………………………………….2

S2. Characteristics of the BISNC interface with L/S coding………………………………....3

S3. Energy harvesting performance…………………………………………………………..6

S4. Tolerance of speed variation for the 0/1-coding BISNC interface……………………….7

S5. Trend of time intervals for rotation and up/down control………………………………..10

S6. Potential functionalities of the sixteen-direction BISNC interface………………………11

S7. Fabrication process of the stretchable BISNC interface…………………………………12

S1. Optimization of electrode width and spacing

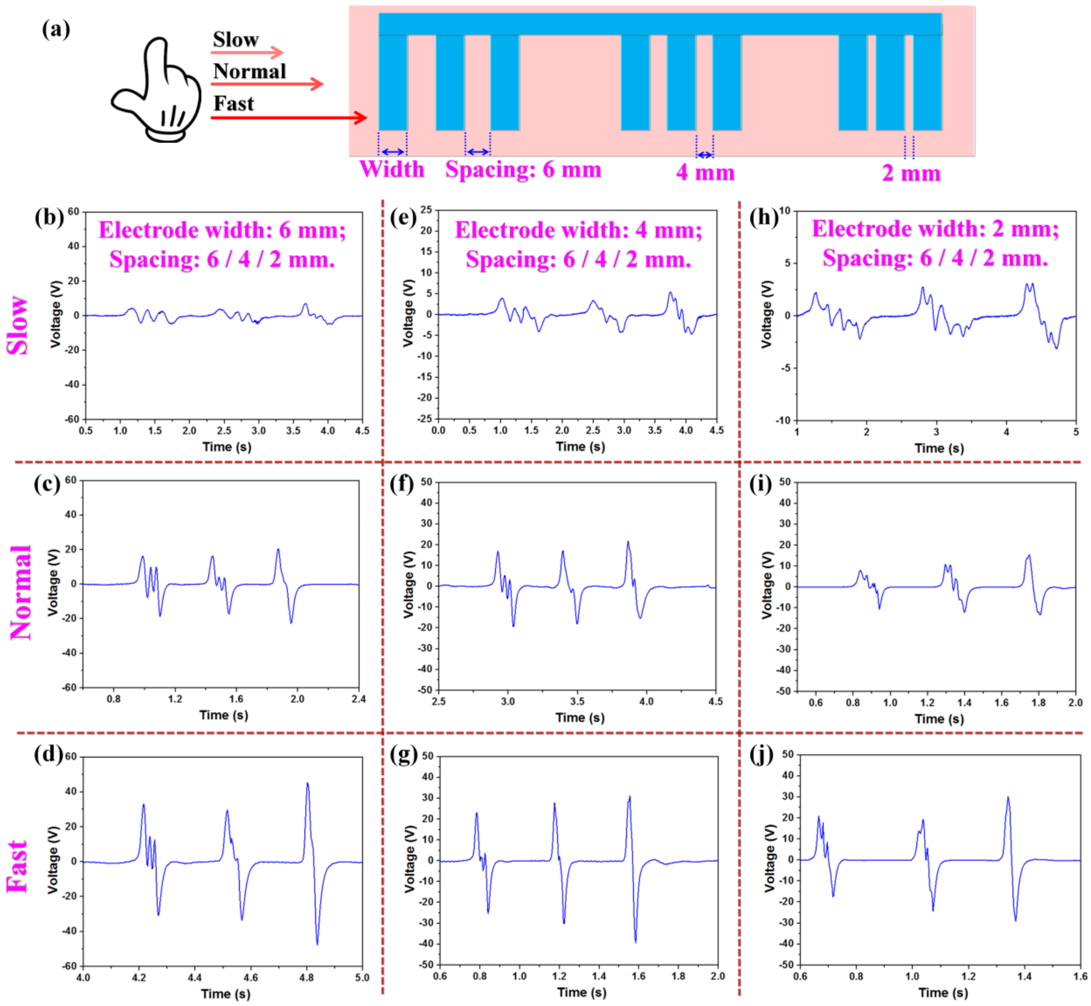

Figure S1. Optimization of the electrode width and spacing. (a) Schematic of electrode patterns with spacing of 6, 4 and 2 mm. (b) Slow, (c) normal, and (d) fast sliding across the electrode patterns with electrode width of 6 mm. (e) Slow, (f) normal, and (g) fast sliding across the electrode patterns with electrode width of 4 mm. (h) Slow, (i) normal, and (j) fast sliding across the electrode patterns with electrode width of 2 mm. To maintain the clear peak identification and miniaturized device size, electrode width of 4 mm and spacing of 6 mm are adopted unless specified.

S2. Characteristics of the BISNC interface with L/S coding

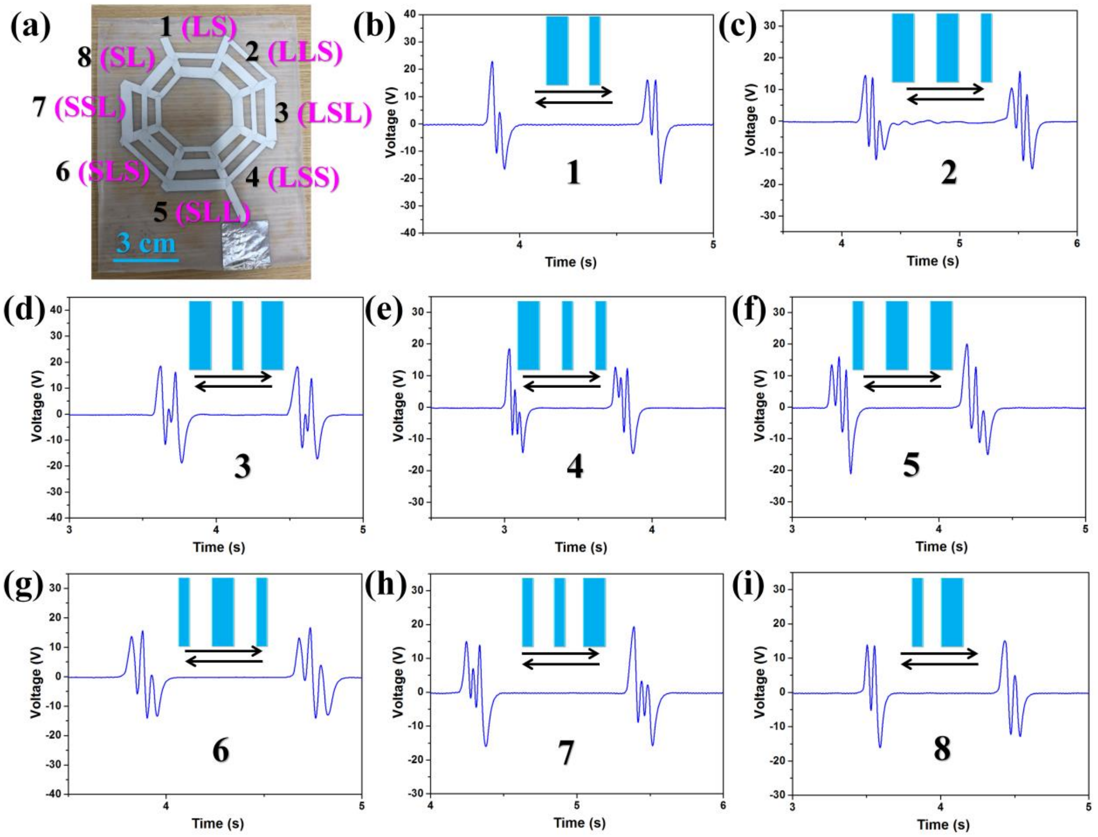

Figure S2. The eight-direction BISNC interface with L/S coding (electrode width of 8 mm and 4 mm, spacing of 6 mm). (a) The photograph of the eight-direction control interface. (b-i) The corresponding generated peaks when finger sliding forward and backward across the eight electrode patterns.

The photograph of a fabricated eight-direction BISNC interface of L/S coding design is shown in Figure S2a, with large electrode width of 8 mm, small electrode width of 4 mm, and electrode spacing of 6 mm. According to the design principle, electrode with larger width should produce higher output peak due to the larger contact area during sliding. In the

measurement, the sliding motion is performed by finger with normal speed to slide forward (inside toward outside) and backward (outside toward inside) across the electrode patterns. The reason for backward sliding is because it is difficult to decode all the directions only based on forward sliding generated peaks with small spacing between the electrodes.

Due to the relatively large size of human finger of ~15 mm and the small electrode spacing of 6 mm, there are situations where finger are covering two electrodes (e.g., finger sliding out of the first electrode and sliding in the second electrode) at the same time. This causes the overlapping of negative component of the first peak and positive component of the second peak, leading to a reduction in amplitude for the second peak, as shown in Figure S2b-i. Due to the overlapping of output peaks, the output signal pattern may not accurately follow the electrode pattern in an ideal case (i.e., larger output signal peak from electrode with larger width and smaller output signal peak from electrode with smaller width).

Therefore, a forward/backward sliding and a detection strategy are proposed for the interpretation of the signal peaks corresponding to the electrode pattern, as illustrated in Table 1. The last two columns of the table indicate the comparison results of the current peak with the former peak (i.e., the second peak with the first peak, and the third peak with the second peak). The comparison results are roughly categorized into three classes, i.e., larger ("L"), equivalent ("E") and smaller ("S"). With the overlapping effect, the large/small (former electrode is large and current electrode is small) comparison result is always "S", same as the electrode pattern. Similarly, results of large/large and small/small can be "E" or "S", while results of small/large comparison can be "L" or "E". For the patterns with only two strip

electrodes (direction 1 and 8), only one comparison is required for forward and backward sliding. Based on the comparison results as indicated in the table, they can be easily differentiated. For the other six directions with three strip electrode in the pattern, whenever an “L” comparison result appears in the forward and backward sliding, it means that the current electrode must have a larger electrode width than the former one. That is, the current electrode width is large, and the former electrode width is small. If both the forward sliding and backward sliding have “L”, the electrode pattern can be easily interpreted, such as direction 3 and 6. Then the other directions can be interpreted according to the comparison results in the table S1.

Table S1. Signal interpretation table (comparison result of the current peak amplitude with the former one: “L” – larger, “E” –equivalent, “S” – smaller).

**Direction**	**Electrode patterns in forward direction**	**Forward sliding (→)**	**Backward sliding (←)**
Direction-1	Large; Small	-S	-E
Direction-2	Large; Large; Small	-ES	-LS
Direction-3	Large; Small; Large	-SL	-SL
Direction-4	Large; Small; Small	-SS	-SL
Direction-5	Small; Large; Large	-LS	-SS
Direction-6	Small; Large; Small	-LS	-LS
Direction-7	Small; Small; Large	-SL	-SS
Direction-8	Small; Large	-E	-S

S3. Energy harvesting performance

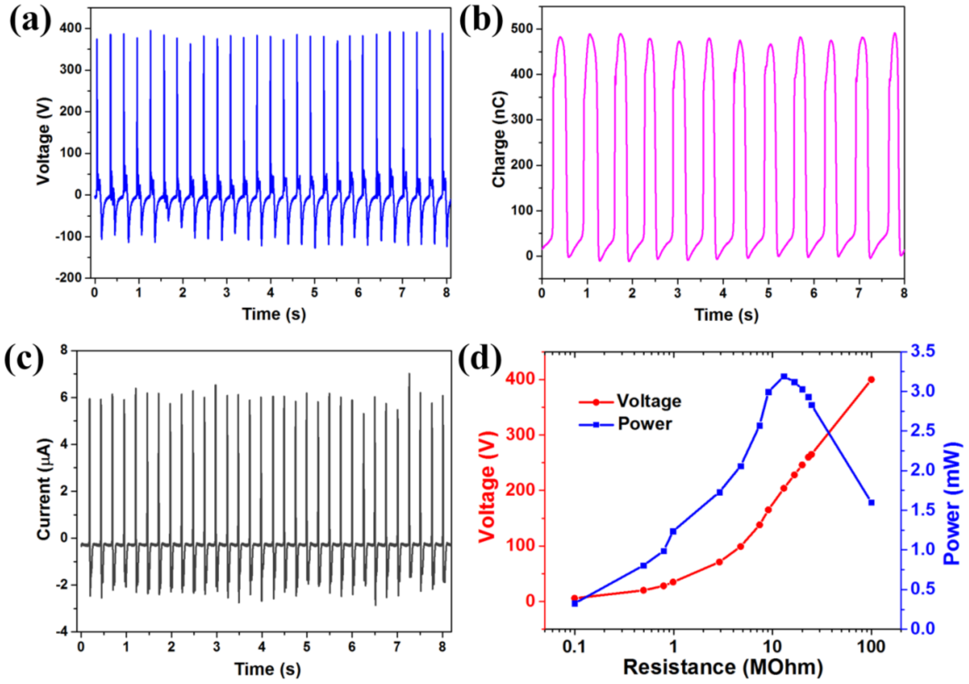

Figure S3. Energy harvesting performance from periodic hand tapping. (a) The generated output voltage on a 100 MΩ load, (b) output charge, and (c) short circuit current. (d) The output voltage and power of the device under different external loads.

S4. Tolerance of speed variation for the 0/1-coding BISNC interface

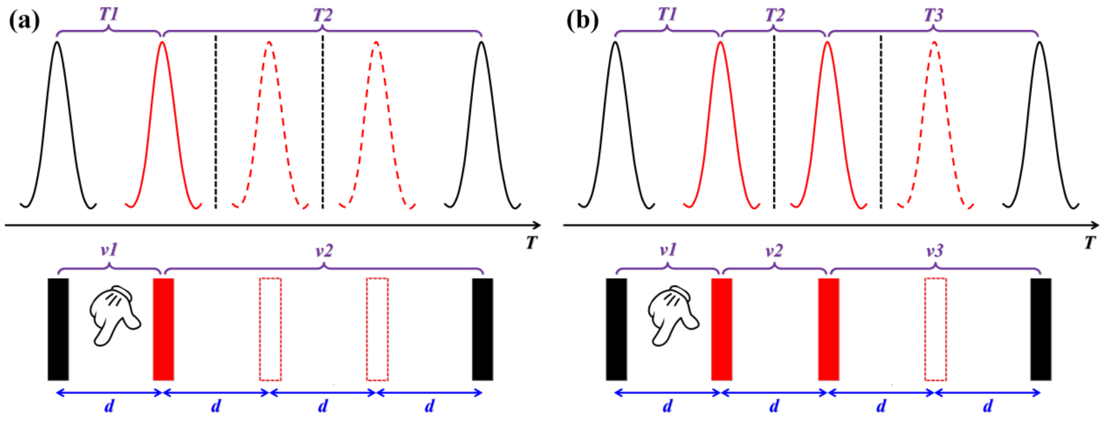

Figure S4. Tolerance of variation in sliding speed for the 0/1-coding BISNC interface. (a) The generated signals for the case of 3 peaks (100). Only the positive peak is illustrated here for simplification. (b) The generated signals for the case of 4 peaks (110).

The 0/1-coding BISNC interface shows excellent robustness and reliability in diverse ambient conditions and usage scenarios, such as different humidity, different sliding force, and different sliding speed, etc. The solo constraint is that the sliding speed should be maintained relatively constant during the sliding, whether in a slow, normal or fast manner, due to the detection mechanism of peak positions in time domain. Therefore, the discussion here is to decide the variation tolerance of sliding speed for practical usage.

There are eight electrode patterns corresponding to the eight directions. Including the beginning and ending reference electrodes, there are 4 classes of generated signals in terms of the number of peaks, i.e., 2 peaks (000), 3 peaks (001, 010, 100), 4 peaks (110, 101, 011), and 5 peaks (111). In the case of 2 peaks and 5 peaks, there is only one possibility for each case,

thus 000 and 111 can be easily recognized based on the number of the generated peaks, irrelevant of the variation in sliding speed.

In the case of 3 peaks, the ideal signal (100) with constant speed is shown in Figure S3a, with black peak representing the references peak, solid red peak representing the real coding peak and dash red peak representing the virtual coding peak generated from the corresponding electrodes. In the schematic, d is the distance between two adjacent electrodes, $v1$ is the average sliding speed from the beginning reference peak to the signal peak, $v2$ is the average sliding speed from the signal peak to the ending reference peak, $T1$ and $T2$ are the time duration. The dash lines in the figure denote the range of the generated signal peak for correct recognition. For 100, $T1$ should be less than 3/8 of the entire time duration between the two reference peaks. After the calculation, the relationship of $v1$ and $v2$ can be achieved, i.e., $v2 < 9/5 \cdot v1$. For 010, $T1$ should locate in between 3/8 and 5/8 of the entire time duration, thus $3/5 \cdot v1 < v2 < 5/3 \cdot v1$. Similarly, for 001, $T1$ should be larger than 5/8 of the entire time duration, thus $v2 > 5/9 \cdot v1$. In summary, the variation of sliding speed should satisfy the condition of $3/5 \cdot v1 < v2 < 5/3 \cdot v1$.

In the case of 4 peaks, the ideal signal (110) with constant speed is shown in Figure S3b. For 110, to achieve correct recognition, first $T3$ should be larger than both $T1$ and $T2$, and then it should be also larger 3/8 of the entire time duration. Thus the following conditions can be achieved, i.e., $v3 < 2 \cdot v1$, $v3 < 2 \cdot v2$, and $v3 < 10/3 \cdot v1 \cdot v2/(v1+v2)$. Similarly, the conditions for 101 and 011 can also be achieved, $v2 < 2 \cdot v1$, $v2 < 2 \cdot v3$, $v2 < 10/3 \cdot v1 \cdot v3/(v1+v3)$, and $v1 < 2 \cdot v2$, $v1 < 2 \cdot v3$, $v1 < 10/3 \cdot v2 \cdot v3/(v2+v3)$. In other words, the

average sliding speed across the virtual electrode should be less than a certain value in order to achieve correct recognition. If we consider $v1 = v2$, then $v3 < 5/3 \cdot v1$. That is, the variation of sliding speed should be less than 166.7% of the original speed.

Therefore, by considering all the scenarios and conditions, the variation of sliding speed should within the range from 60% to 166.7%, in order to achieve correct recognition. That is, the 0/1-coding control interface has a variation tolerance in sliding speed of at least ±40%.

S5. Trend of time intervals for rotation and up/down control

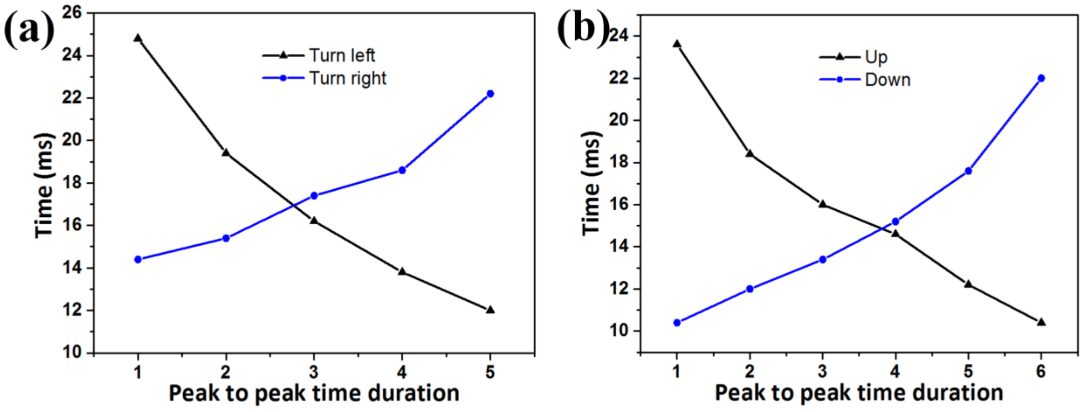

Figure S5. Time intervals when sliding across the two additional sensing patterns in the eight-direction BISNC interface with 0/1 coding. (a) The peak-to-peak time interval in the generated signals for sliding left and right. (b) The peak-to-peak time interval in the generated signals for sliding up and down.

S6. Potential functionalities of the sixteen-direction BISNC interface

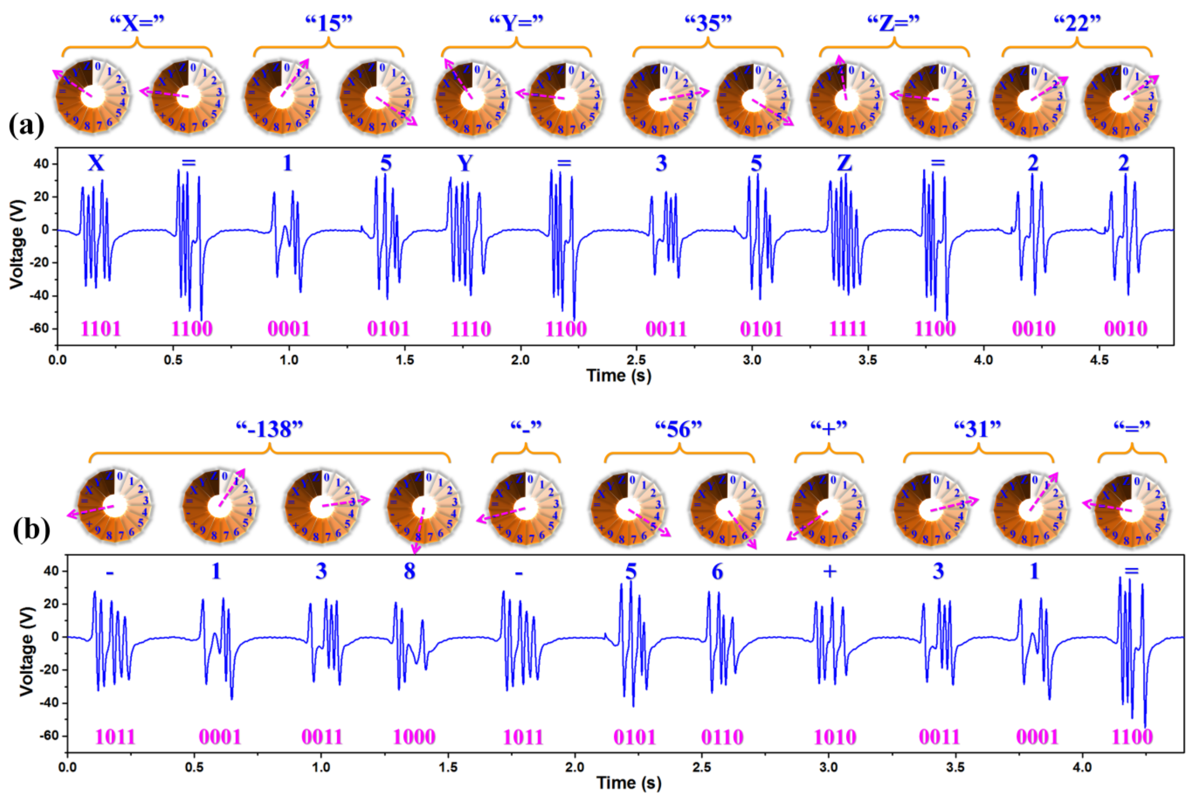

Figure S6. Functional BCD interface. (a) Setting the spatial coordinates of point (15, 35, 22). (b) Inputting “-138-56+31=” for calculation.

S7. Fabrication process of the stretchable BISNC interface

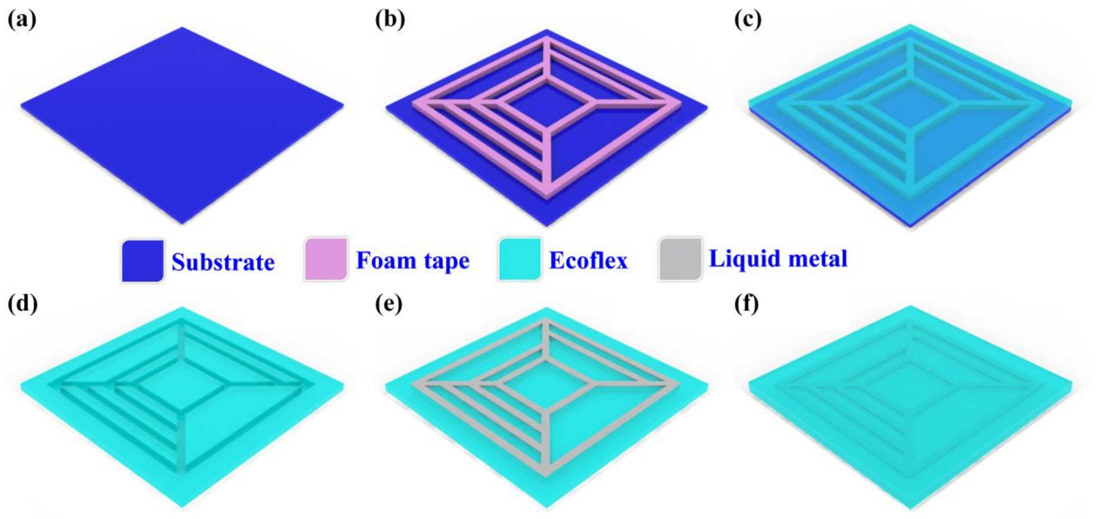

Figure S7. Fabrication process of the stretchable BISNC interface. (a) Substrate preparation. (b) Fluidic channels forming by foam tape. (c) Ecoflex moulding. (d) Ecoflex peeling off with fluidic channels. (e) Liquid metal filling into the channels. (f) Pouring and curing of another Ecoflex layer for encapsulation.

Supporting Videos

Video S1. Demonstration of 3D drone control.

Video S2. Operation of the flexible BISNC interface.

Video S3. Operation of the stretchable BISNC interface.
